# *Leuconostoc citreum* TR116 as a Microbial Cell Factory to Functionalise High-Protein Faba Bean Ingredients for Bakery Applications

**DOI:** 10.3390/foods9111706

**Published:** 2020-11-20

**Authors:** Andrea Hoehnel, Jürgen Bez, Aylin W. Sahin, Aidan Coffey, Elke K. Arendt, Emanuele Zannini

**Affiliations:** 1 School of Food and Nutritional Sciences, University College Cork, College Road, T12K8AF Cork, Ireland; andrea.hoehnel@umail.ucc.ie (A.H.); aylin.sahin@ucc.ie (A.W.S.); e.zannini@ucc.ie (E.Z.); 2 Fraunhofer Institute for Process Engineering and Packaging, 85354 Freising, Germany; juergen.bez@ivv.fraunhofer.de; 3 Department of Biological Sciences, Cork Institute of Technology, T12K8AF Cork, Ireland; aidan.coffey@cit.ie; 4 APC Microbiome Ireland, T12K8AF Cork, Ireland

**Keywords:** plant-protein, legumes, faba bean, lactic acid fermentation, phenolic acids, baking properties, GlutoPeak, bread quality

## Abstract

Grain legumes, such as faba beans, have been investigated as promising ingredients to enhance the nutritional value of wheat bread. However, a detrimental effect on technological bread quality was often reported. Furthermore, considerable amounts of antinutritional compounds present in faba beans are a subject of concern. Sourdough-like fermentation can positively affect baking performance and nutritional attributes of faba bean flours. The multifunctional lactic acid bacteria strain *Leuconostoc citreum* TR116 was employed to ferment two faba bean flours with different protein contents (dehulled flour (DF); high-protein flour (PR)). The strain’s fermentation profile (growth, acidification, carbohydrate metabolism and antifungal phenolic acids) was monitored in both substrates. The fermentates were applied in regular wheat bread by replacing 15% of wheat flour. Water absorption, gluten aggregation behaviour, bread quality characteristics and in vitro starch digestibility were compared to formulations containing unfermented DF and PR and to a control wheat bread. Similar microbial growth, carbohydrate consumption as well as production of lactic and acetic acid were observed in both faba bean ingredients. A less pronounced pH drop as well as a slightly higher amount of antifungal phenolic acids were measured in the PR fermentate. Fermentation caused a striking improvement of the ingredients’ baking performance. GlutoPeak measurements allowed for an association of this observation with an improved gluten aggregation. Given its higher potential to improve protein quality in cereal products, the PR fermentate seemed generally more promising as functional ingredient due to its positive impact on bread quality and only moderately increased starch digestibility in bread.

## 1. Introduction

Many dietary recommendations as well as guidelines to improve sustainability along the food chain advise a drastic decrease in animal proteins in our diets and an enhanced inclusion of plant-based protein sources [1,2,3]. Even though cereal products, such as the staple food bread, can be considered an important source of plant-based protein in human diets worldwide, its poor protein quality and relatively low protein content make it an inadequate choice to (at least partially) compensate for substantially decreased animal protein intakes in our diets. The poor quality of wheat protein is primarily related to an unbalanced amino acid profile and insufficient contents of indispensable amino acids, specifically lysine [4,5,6]. Furthermore, the high content of rapidly digestible starch causing a high glycaemic index (GI)/glycaemic load (GL) of white wheat bread has been mentioned as one of the rather disadvantageous traits [6,7,8]. Jenkins et al. [8] mentioned that foods should possess nutritional rather than nutrient density (referring to an increased intake of essential macro- and micronutrients per calorie); especially in the context of generally decreased physical activity in our modern society. The partial substitution of wheat flour in bread formulations by various plant-based protein ingredients (cereals, pseudocereals, potato, vegetable and legumes) with the objective to improve bread nutritional quality, and primarily protein content and quality, has been thoroughly investigated within the past two decades [9,10,11,12,13,14,15]. Boukid et al. [16] reported that specifically grain legumes, such as faba beans, are very promising for this approach. Besides their promising environmental foot print and beneficial nitrogen fixing properties during cultivation [17,18], they also provide an amino acid profile that offers a matching complementation to that of wheat [12,16,19]. While a substantial improvement of the overall nutritional quality of the breads, with an emphasis on protein quality, was often achieved, many studies also reported severely decreased technological bread quality. Specifically higher levels of wheat flour replacement, which are chosen to enhance the potential nutritional improvement, seem to pose major challenges. Therefore, plant-based high-protein ingredients (HPIs) are particularly promising as they provide higher amounts of protein and a higher capacity to compensate for the lack of certain amino acids at lower levels of wheat flour substitution. Dry fractionated (air-classified) legume flours, for example, offer a substantially increased protein content while their production is less energy and resource-intensive [18,20]. Another concern, besides drastically reduced technological bread quality, involves antinutritional compounds (ANCs) which occur in legumes and can, amongst other effects, negatively impact absorption of minerals and protein digestibility [21]. For the improvement of the baking performance of faba bean flour and to reduce its contents of ANCs fermentation technology, and specifically sourdough-like single-strain fermentation, has been investigated and described to be a powerful tool [22,23,24,25,26,27,28]. While various lactic acid bacteria (LAB) strains promise a positive impact, specifically *Leuconostoc* species have been reported to produce or release a large range of compounds with biological activities and potentially beneficial techno-functional properties [29,30,31,32]. The strain *Leuconostoc citreum* TR116 is a multifunctional LAB strain and has, for example, been found to efficiently convert fructose present in the substrate into mannitol [33]. Fermentates produced with this strain have successfully been applied in beverages and bakery products to reduce contents of added sugar while maintaining adequate sensory characteristics [34,35,36,37]. In the present study, *Ln. citreum* TR116 is applied to ferment two faba bean (FB) ingredients: FB dehulled flour (DF) and air-classified FB high-protein flour (PR). The strain’s fermentation profile in a low- and high-protein environment originating from the same raw material is thereby compared. The produced sourdough-like fermentates are used to replace 15% (based on solids) of wheat flour in a regular wheat bread formulation. The results are compared to those of breads containing the same level of unfermented FB ingredients and to a control wheat bread. The objective of this study is to assess the potential of faba bean fermentation with *Ln. citreum* TR116 to create a functional fermented HPI for bakery applications. The study approach combining fundamental characterisation of faba bean fermentation with a detailed investigation of the application of the produced fermentates in breads promises high relevance of the results for advancing the protein transition.

## 2. Materials and Methods

### 2.1. Materials

Two different ingredients produced from faba beans (FB; *Vicia Faba* L.) were used in this study: faba bean dehulled flour (DF; protein content (N × 6.25) 33.94 %DM, fat 1.15 %DM, ash 3.28 %DM, fibre 10.18 %DM, carbohydrates by difference 62.00 %DM, total starch 47.37 %DM) and faba bean high-protein flour (PR; protein content (N × 6.25) 61.25 %DM, fat 3.81 %DM, ash 5.43 %DM, fibre 0.35 %DM, carbohydrates by difference 29.17 %DM, total starch 7.77 %DM [11]; obtained by dry fractionation), which were experimentally produced and provided by Fraunhofer Institute IVV, Freising, Germany. For the preparation of sourdough-like faba bean fermentates by controlled single-strain fermentation, the lactic acid bacteria (LAB) strain *Ln. citreum* TR116 was used, which was originally isolated from yellow pea sourdough. This strain belongs to the culture collection of the Department of Biological Sciences, Cork Institute of Technology. Conditions for the cultivation and storage were according to those reported by Sahin et al. [35]. For baking trials, wheat flour was supplied by Whitworth Bros Ltd., Wellingborough, UK; dry yeast (4% moisture, 50% protein, 5% fat, 40% carbohydrates) by Puratos, Dilbeek, Belgium; salt by Glacia British Salt Ltd., Northwich, UK; and vegetable oil by Musgrave, Cork, Ireland. Chemicals were purchased from Sigma-Aldrich (St. Louis, MO, USA) unless stated otherwise.

### 2.2. Production of Sourdough-Like Fermentates

To obtain a cell suspension of TR116, a single colony was transferred into 10 mL of de Man–Rogosa–Sharpe (MRS) broth and incubated at 30 °C for 24 h. This preinoculum was subcultured (100 μL in 10 mL MRS broth) and incubated at 30 °C for 16 h. The cells were harvested by centrifugation (4800 *g*, 10 min, 10 °C), washed twice with sterile tap water (addition of 10 mL tap water, resuspension and subsequent centrifugation with same conditions as above) and finally resuspended in 10 mL of sterile tap water and used as inoculum. The desired dough yield of 200 (dough yield; DY = 100 × (flour weight [g] + water weight [g])/(flour weight [g])) was achieved by mixing the respective faba bean ingredient and liquid (sterile tap water and cell suspension, volume calculated to reach an initial cell density of 7 log CFU (cell forming units)/g) in a ratio of 1:1 with a Kenwood Chef (Kenwood Manufacturing Co. Ltd., Havant, UK) kitchen machine equipped with a K-beater attachment for 1 min at speed 1, followed by 1 min at speed 2. Aliquots of the mixture were filled into separate sterile stomacher bags to enable analysis of the sourdough-like fermentates at different time points throughout the fermentation process. The stomacher bags were airtightly sealed and incubated at 30 °C for a maximum of 48 h. The presented results were obtained from three independently performed fermentation trials for each of the two faba bean ingredients. The sourdough-like fermentates produced from DF and PR are further referred to as DFSD and PRSD, respectively.

### 2.3. Characterisation of Fermentation Profile

To characterise and compare the fermentation profiles of *Ln. citreum* TR116 in the two different faba bean substrates, the fermentation process was monitored and fermentates were analysed every 6 h (starting at 0 h) over a total fermentation time of 48 h with respect to microbial growth, acidification, carbohydrate levels and contents of organic acids with antifungal activity. After sample collection at the respective time points, samples were either analysed fresh (cell count, pH, total titratable acids (TTA)) or immediately frozen for further processing and extraction.

#### 2.3.1. Determination of Cell Count, pH and Total Titratable Acids

Microbial growth and acidification of the samples were monitored by measuring the cell count, pH and TTA. These analyses were performed with the fresh fermentates after sample collection from the incubator. Cell counts were determined by diluting 1 g of fermentate with 9 mL sterile Ringer’s solution and preparing a decimal dilution series as described by Sahin et al. [35]. Dilutions were plated on MRS agar containing 0.05 g/L bromocresol green and incubated anaerobically for 48 h at 30 °C. The cell count was calculated based on the counted colonies using the weighted arithmetic average. The pH and TTA of the samples were measured according to AACC 02–31.01 [38] in a duplicate for each time point of each of the three fermentation trials.

#### 2.3.2. Preparation of Fermentates for Extraction

The frozen fermentate samples were mechanically crushed into smaller fragments and transferred into 50 mL test tubes. The samples were frozen at −80 °C for a minimum of 30 min and subsequently freeze-dried. The dry fermentates were ground to a fine powder with a mortar and pestle and stored frozen in sealed 50 mL test tubes. For the calculation of results based on dry matter, the moisture of the freeze-dried fermentates was determined with the air-oven method (AACC method 44–15.02 [39]). The extractions described below were carried out in a duplicate for each time point of each of the three fermentation trials.

#### 2.3.3. Levels of Organic Acids and Carbohydrates

For the quantification of organic acids and mono-, di- and oligosaccharides and polyols, the freeze-dried fermentates were extracted according to the procedure previously described by Hoehnel et al. [12] with some modifications. In brief, 1 g of sample was dispersed in 15 mL of 80/20 (*v*/*v*) ethanol/ultrapure water (80% EtOH), with a temperature of 55 ± 5 °C. The extraction was facilitated by a sonication step followed by centrifugation and separation of the supernatant from the solids. The extraction procedure was repeated with the pellet from step 1 and the supernatants from both extraction steps were pooled. A vacuum centrifuge was used to evaporate ethanol and to concentrate the extracts before the final volume was adjusted to 10 mL with ultrapure water containing 50 mg/L NaN${}_{3}$. The obtained fermentate extracts were filtered through 0.2 μm syringe-driven filters and stored frozen in sealed 1.5 mL screw cap tubes until chromatographic analysis. The quantification of selected mono-, di-, and oligosaccharides and polyols (sucrose, glucose, fructose, mannitol, verbascose, stachyose/raffinose, galactose) was carried out as previously described by Ispiryan et al. [40] with a Dionex ICS-5000+ system (Sunnyvale, CA, USA) and pulsed amperometric detection. An external calibration was used for each of the compounds (0.1–20 ppm) and the separation was performed on two different columns: a Thermo Scientific Dionex CarboPac PA1 column (2 × 250 mm; Thermo Fisher Scientific, Waltham, MA, USA; used for quantification of sucrose, glucose, fructose, mannitol, galactose) and a Dionex CarboPac PA200 column (3 × 250 mm; Thermo Fisher Scientific, Waltham, MA, USA; used for quantification of verbascose, stachyose/raffinose). Both columns were equipped with the matching guard column. Flow rate, eluents and elution conditions (isocratic/gradient) were as specified by Ispiryan et al. [40]. Other mono-, di- and oligosaccharides such as arabinose, xylose, maltose and maltotriose were included in the screening but not detected in quantities higher than 0.02 %DM (throughout the fermentation process until 48 h of fermentation) and therefore not included in the reported results. The separation of acetic acid and lactic acid was performed using a Dionex UltiMate 3000 System (Thermo Fisher Scientific, Waltham, MA, USA) equipped with a Hi-Plex H column (7.7 × 300 mm; Agilent Technologies, Santa Clara, CA, USA) and the matching guard column, which was operated at a column temperature of 60 °C and with 5 mM sulphuric acid as eluent. The compounds were quantified using an external calibration (0.03–6 g/L) and a ultraviolet light/diode array detector (UV/DAD; Thermo Fisher Scientific, Waltham, MA, USA; UV spectra recorded between 190 and 400 nm; quantification at 210 nm). Additionally, the fermentation quotient, which represents the molar ratio of lactic and acetic acid (lactic acid [mmol/L]/acetic acid [mmol/L]), was calculated.

#### 2.3.4. Level of Antifungal Compounds

The fermentates DFSD and PRSD were screened for the presence of 15 phenolic compounds with previously reported antifungal activity [41,42,43,44] (catechol, 4-hydroxyphenyllactic acid, 4-hydroxybenzoic acid, hydrocaffeic acid, vanillic acid, caffeic acid, phloretic acid, 3-phenyllactic acid, hydroferulic acid, *p*-coumaric acid, ferulic acid, benzoic acid, salicylic acid, hydrocinnamic acid, methylcinnamic acid). The extraction of these compounds was carried out based on the QuEChERS (quick, easy, cheap, effective, rugged and safe) procedure described by Brosnan et al. [45] with some modifications. Briefly, 1 g of the freeze-dried fermentates was dispersed in 10 mL of ultrapure water, followed by the addition of 10 mL of ethyl acetate containing 0.1% (*v*/*v*) formic acid and homogenisation using a vortex shaker. Subsequently, 1 g NaCl and 4 g MgSO${}_{4}$ were added and the samples were thoroughly shaken by hand for one minute. The organic phase of the supernatants of the extracts was obtained and separated after centrifuging at 4800 *g* for 10 min and transferred into solid-phase extraction (SPE) tubes (Bond Elut QuEChERS Dispersive kit; Agilent Technologies Inc., Santa Clara, CA, USA). The tube contents were first thoroughly homogenised and then centrifuged at 2300 *g* for 10 min. Five millilitres of the obtained supernatant was added to 100 μL dimethylsulfoxide (DMSO) and concentrated using a vacuum centrifuge (Scanvac, Scanspeed; 2 h, 500 rpm, 45 °C). The concentrated extracts were reconstituted by adding 400 μL 90/10 (*v*/*v*) ultrapure water/acetonitrile and filtered through 0.2 μm syringe driven filters before chromatographic analysis. The separation and quantification of antifungal compounds was performed on a Dionex UltiMate 3000 RSLC system (Thermo Fisher Scientific, Waltham, MA, USA) equipped with a a Gemini C18 column (2 × 150 mm; Phenomenex Inc., Torrance, CA, USA), matching guard cartridge and a UV/DAD detector (Thermo Fisher Scientific, Waltham, MA, USA; UV spectra recorded between 190 and 400 nm; quantification at 210 nm). The column temperature was set to 30 °C and the following gradient elution based on two eluents (eluent A-ultrapure water containing 0.1% formic acid; eluent B-acetonitrile containing 0.1% formic acid) was applied at a constant flow rate of 0.2 mL/min: (1) 0–5 min, 95% A; (2) 5–15 min, 95–85% A; (3) 15–35 min, 85–60% A; (4) 35–45 min, 60–50% A; (5) 45–50 min, 50–5% A; (6) 50–70 min 95% A. An external calibration (5–50 ppm) was used to quantify antifungal compounds. Only five (4-hydroxybenzoic acid, caffeic acid, coumaric acid, ferulic acid, phenyllactic acid) of the 15 compounds were detected in levels above the limit of quantification (5 mg/100 g DM). Therefore, only results of these five compounds are presented in this work.

### 2.4. Application of Sourdough-Like Fermentates and Product Analysis

For the application of DFSD and PRSD in bread (and for the determination of flour properties), the maximum fermentation time of 48 h was chosen. Five different bread formulations were prepared: a control wheat bread formulation based on wheat flour (CWB), two faba bean bread formulations with partial replacement of wheat flour by unfermented faba bean ingredients (DFB and PRB) and two faba bean bread formulations with the sourdough-like faba bean fermentates (DFSDB and PRSDB). The fermentates used for both flour analysis and baking trials were stored at 4 °C (for a maximum of 2 h) after sample collection and conditioned in a water bath at 25 °C for 10 min immediately before use.

#### 2.4.1. Flour Analysis

The properties of wheat flour (used for CWB) and FB flour mixtures (DF flour mix, DFSD flour mix, PR flour mix and PRSD flour mix used for DFB, PRB, DFSDB and PRSDB, respectively) were analysed with regard to water absorption and gluten aggregation behaviour. In FB flour mixtures, 15% of wheat flour was replaced with the respective faba bean ingredient (equivalent to the flour mixtures used for baking trials, see Table 1). For DFSD flour mix and PRSD flour mix, 15% of wheat flour were replaced by the fermentates based on their solid fraction (50% due to the chosen DY of 200). The liquid fraction that was brought into DFSD flour mix and PRSD flour mix by the fermentates was accounted for when recipes and results for the Farinograph and GlutoPeak measurements were calculated. Moreover, the moisture content of the FB flour mixtures was calculated based on the moisture content determined for the raw materials (wheat flour, DF and PR) and their ratios. The weigh-in of wheat flour and FB flour mixtures for each test was adjusted to match a theoretical moisture content of 14% according to AACC 82–23.01. The determination of Farinograph (Brabender GmbH and Co KG, Duisburg, Germany) water absorption (FWA) was performed according to AACC 54–21.02. Titration trials were performed in a triplicate adjusting doughs from wheat flour and FB flour mixtures to a consistency of (500 ± 20) Farinograph units (FU). Subsequently, a full Farinograph measurement (over 20 min) with the initial water addition set to the mean FWA determined from titration trials was performed to confirm the previous results. The obtained FWAs for DFSD and PRSD flour mixtures were corrected for the water amount originating from the fermentates. The gluten aggregation properties of wheat flour and FB flour mixtures were evaluated according to the procedure previously reported by Heohnel et al. [11] with a GlutoPeak device (Brabender GmbH and Co KG, Duisburg, Germany). Briefly, a flour/water slurry (50:50 ratio; adjusted based on AACC 82–23.01 and subtracting water from fermentates) was prepared and the following test settings were applied: 36 °C; paddle speed 2750 rpm. The test variables torque maximum (TM, expressed in Brabender units BU) and peak maximum time (PMT, expressed in s) were retrieved from the test and are presented in addition to gluten aggregation profiles.

#### 2.4.2. Recipe Adaptation and Bread Production

Bread samples were prepared according to the procedure described by Hoehnel et al. [11]. For incorporation of FB ingredients (DF, PR, DFSD, PRSD), 15% of wheat flour was replaced (for DFSD and PRSD, based on solid fraction of fermentates). Water levels where applied as determined by Farinograph trials FWAs and adjusted for the water originating from fermentates incorporated in DFSDB and PRSDB formulation (Table 1). The presented results represent the mean of three independently performed baking trials; in the case of DFSDB and PRSDB with fermentates prepared in three independent fermentation trials.

#### 2.4.3. Determination of Dough pH

The pH of bread doughs was analysed with the same method as for FB fermentates (described above). After mixing, dividing and moulding, the dough was stored at 4 °C (for a maximum of 5 h) until analysis. An Ultra Turrax (IKA Werke, Staufen, Germany) was used to homogenise the samples.

#### 2.4.4. Bread Quality Analysis

Analysis of bread quality characteristics was performed according to the procedures and test settings reported by Hoehnel et al. [11]. The following bread quality characteristics have been determined for the breads produced in this study; bake loss (gravimetrically by weighing dough after shaping and bread loaf after cooling down), specific volume (SV; with a Volscan Profiler; Stable Micro Systems, Godalming, UK), crumb hardness (on days 0, 2 and 5 of storage; with a TA-XT2i Texture Analyser; Stable Micro Systems, Godalming, UK), crumb structure (with a C-Cell Imaging System; Calibre Control International Ltd, Warrington, UK) and lightness of crust and crumb (Colorimeter CR-400; Konica Minolta, Tokyo, Japan). Based on the crumb hardness determined on days 0 and 5 of storage, the staling rate of all five bread formulations was calculated with the following equation.

(1) $$Staling\;\;rate=\frac{crumb\;\;hardness\;\;day\;\;5-crumb\;\;hardness\;\;day\;\;0}{crumb\;\;hardness\;\;day\;\;0}$$

The values obtained for lightness of crust (L ${}^{*}$ crust) and lightness of crumb (L ${}^{*}$ crumb) are reported using the CIE L ${}^{*}$a ${}^{*}$b ${}^{*}$ colour space.

#### 2.4.5. Scanning Electron Microscopy

Scanning electron microscopy (SEM) of bread samples was performed according to the procedure described by Hoehnel et al. [12]. The samples were coated with a 5 nm layer of gold–palladium (80:20) and subsequently examined under the microscope; images were acquired at a constant accelerating voltage of 5 kV.

### 2.5. In Vitro Starch Digestion

In vitro starch digestion was performed to monitor the release of reducing sugars over time and to determine the hydrolysis index (HI) value. This value is calculated by dividing the area under the sugar release curve (30 to 240 min) of a certain sample (DFB, DFSDB, PRB and PRSDB in this study) by the area under the sugar release curve (30 to 240 min) of a reference/control sample (CWB in this study). The experiments were carried out using the bread crumb from defrosted bread samples and following the procedure reported by Brennan and Tudorica [46] with some modifications. The samples were subjected to a pepsin treatment (pH 1.5) followed by an incubation with pancreatic α-amylase (pH 6.9) for five hours in a dialysis tube. The release of reducing sugars into the buffer surrounding the dialysis tubing was monitored (sample collection every 30 min) and the content of reducing sugars in the samples was determined spectrophotometrically using 3,5-dinitrosalicylic acid (DNS; 10 g/L) as colouring reagent (100 μL of DNS solution was added to 100 μL of sample, the mixture was further diluted with 1 mL buffer and then incubated at 110 °C for 15 min before absorbance was read at 546 nm). Appropriate blanks (sample blank with deactivated enzymes; spiked sample blank with deactivated enzymes and added maltose; maltose blank) were run with the samples in order to evaluate whether differences in the release of reducing sugars are caused by an altered diffusion rate of reducing sugars or possibly a delayed/reduced starch degradation. The amount of released reducing sugars was expressed as percentage of the digestible starch present in the sample. Contents of resistant and digestible starch were determined using the enzyme kit K-RAPRS (Megazyme, Ireland) and freeze-dried samples of bread crumb.

### 2.6. Statistical Analysis

Both fermentation and baking trials were performed in triplicate. For the characterisation of fermentation profiles, measurements of cell count, pH, TTA, acids, sugars and antifungal compounds were performed in duplicate for each of the three independent fermentation trials. In vitro starch digestibility of bread samples was analysed in duplicate. Results are presented as the mean ± standard deviation. Data analysis was carried out using RStudio, version 1.2.1335 with R version 3.6.1 (RStudio Inc, Boston, MA, USA; R Core Team, r-project). A correlation analysis (CA, significance level *p* < 0.05) was performed to determine correlation coefficients (r) for results of bread quality characteristics. One-way analysis of variance (ANOVA) with post hoc pairwise Tukey’s test was used to show significant differences (*p* < 0.05) between groups for data from both fermentation and baking trials. In addition, a two-way ANOVA was performed for fermentation characteristics in order to determine the influence of the substrate (DF/PR) and the fermentation time as well as the interactive effect of both of these factors.

## 3. Results and Discussion

### 3.1. Fermentation Profile

The growth and fermentation characteristics of *Ln. citreum* TR116 were monitored for a sourdough-like fermentation of two different FB ingredients over a fermentation time of 48 h.

#### 3.1.1. Microbial Growth and Acidification

**Figure 1:**
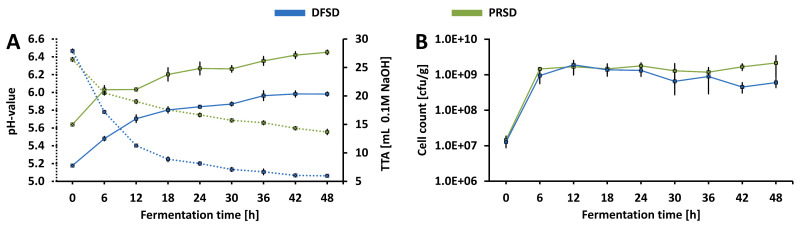
Acidification and microbial growth of *Leuconostoc citreum* TR116 in DFSD and PRSD during 48 h of fermentation: (**A**) Development of pH (represented by dotted lines) and total titratable acids (TTA) (represented by solid lines); (**B**) Determined cell densities.

The pH, TTA and cell count (Figure 1) of the fermentates were determined as key factors describing the fermentation characteristics of LABs in various matrices and media. While the results for pH and TTA reveal major differences in acidification, the cell counts are relatively similar and prove comparable growth of *Ln. citreum* TR116 in both DFSD and PRSD. The initial cell densities (0 h) in the fermentates reflect with 7.11 log CFU/g (DFSD) and 7.16 log CFU/g (PRSD) the inoculation level of 7 log CFU/g. Within the first six hours, cell densities substantially increased by approximately 2 log CFU/g to 8.95 log CFU/g in DFSD and 9.16 log CFU/g in PRSD. This point (6 h) marks the start of a stationary growth phase in both fermentates; no further significant increase in cell density was observed during the remaining fermentation time. While this stationary phase seems to last until the maximum fermentation time of 48 h in PRSD, DFSD shows a tendency towards a decline of growth beginning at 30 h. The determined cell densities in DFSD from 30 h forward are significantly lower than the corresponding cell counts in PRSD (except 36 h; no significant difference detected) as well as the maximum cell density in DFSD at 12 h (9.25 log CFU/g). This could be associated with increased stress and the beginning of a death phase of TR116 growth in DFSD. Sahin et al. [35] reported a similar growth pattern when *Ln. citreum* TR116 was used to ferment wheat flour (same DY and inoculation cell density) for 48 h. However, the stationary phase of growth in wheat flour between 12 and 24 h seems to have been followed by a more pronounced death phase than in DFSD with a decline of cell density by 1.8 log CFU/g (compared to 0.5 log CFU/g in DFSD; calculated as difference to maximum cell density).

The determined pH values in this study indicate a stronger acidification of DFSD than PRSD. While the initial pH at 0 h is similar for both fermentates (6.47 in DFSD and 6.37 in PRSD), this is followed by a much stronger pH drop in DFSD than in PRSD. Especially within the first 12 h, the acidification rate was with 0.09 h ${}^{-1}$ more than twofold higher in DFSD than in PRSD with 0.04 h ${}^{-1}$. After this initial rapid decrease in pH, a more moderate decline was observed between 12 and 48 h of fermentation with the same acidifcation rate of 0.0094 h ${}^{-1}$ in both DFSD and PRSD. While this could have been associated with a higher production of organic acids in DFSD than in PRSD, the contents of acetic and lactic acid, which were additionally determined (Table 2), do not confirm such a correlation. On the contrary, slightly (but significantly; except 6 h) higher levels of acetic acid were produced in PRSD. The levels of lactic acid did not significantly differ (except 12 h) between DFSD and PRSD when the same fermentation time points are compared. This is also evident with regard to the generally slightly lower fermentation quotients obtained for PRSD. The fact that in PRSD both a smaller drop in pH and a slightly higher acid production was observed might be related to a higher buffering capacity. The significantly higher TTA values determined for PRSD support this theory. At all fermentation time points, more 0.1 M NaOH was needed to titrate PRSD fermentates to pH 8.5 than for DFSD fermentates. Considering the higher initial TTA of PRSD at 0 h and the relatively consistent difference of TTAs determined for DFSD and PRSD, it can be assumed that the buffering capacity does rather originate from compounds present in the raw material than from metabolites of TR116. Proteins are, besides peptides, amino acids and organic acids, the most important compounds affecting the buffering capacity in foods [47]. Due to the nearly twofold higher protein content in PR compared to DF, a higher buffering capacity can be expected in PRSD. While in many sourdough-like fermentates the produced organic acids are likely to contribute to buffering capacity, this is expected to have only a minor impact in the fermentates in this study considering the pH ranges within which acetic acid (3.75–5.75) and lactic acids (2.86–4.86) have buffering capacity and the pH values reached in DFSD and PRSD [48]. Furthermore, minerals have been reported to cause increased buffering capacity in food matrices [47]. Therefore, the higher abundance of minerals in PR than in DF (indicated by ash contents: 3.28 %DM in DF; 5.43 %DM in PR) could have further contributed to the elevated buffering capacity in PRSD. Since it is known that acidic environments can cause an inhibition of growth [30] for *Leuconostoc* species, the increased buffering capacity might also be the cause for the persisting stationary growth phase in PRSD by moderating both the pH drop and pH-associated stress for TR116. This is further in line with the previously mentioned findings of Sahin et al. [35] who reported lower pH values as well as a more pronounced decrease in cell density in wheat flour fermented with TR116.

Two-way ANOVA identified a significant impact of both the chosen substrate (DF/PR) as well as the fermentation time on all variables discussed in this section (except FQ; only substrate impact was found sginifcant). The impact of the interaction between substrate and fermentation was found to be significant only for the determined pH values. This is evident with regard to the curves displayed in Figure 1: the substrate influences the way the pH of the fermentates changes over time leading to strikingly different acidification rates within the first 12 h of fermentation as mentioned above. In a study by Coda et al. [22] different air-classified faba bean fractions (faba bean dehulled flour, air-classified protein-fraction; air-classified starch fraction) were fermented with a *Lactobacillus plantarum* strain (VTT E-133328) using the same dough yield, incubation temperature and inoculation cell density as applied in the present study. While the increase in cell density within the first 24 h of fermentation was similar to the growth observed in this study, a significant difference was found between growth in the dehulled flour and the protein fraction (more growth in protein fraction). Moreover, they determined similar changes in pH for these two substrates and generally a more pronounced pH drop (ΔpH at 24 h: 1.85 (dehulled flour) or 1.86 (protein fraction) as opposed to 1.27 (DFSD) and 0.62 (PRSD) in this study). Although it is known that different LAB species show unique growth and acidification profiles in various substrates, a less pronounced pH drop due to increased buffering capacity of the protein fraction, as observed in the present study, could have been expected also for the fermentation with the respective *Lactobacillus plantarum* strain. While the ash content of the protein fraction investigated by Coda et al. [22] was with 5.98 %DM very similar to that of PR in the present study (5.43 %DM), a slightly lower protein content was reported (51.49 %DM in protein fraction in [22] as opposed to 61.25 %DM in PR). Potentially, this higher protein content in combination with the high ash level caused a substantially increased buffering capacity in PRSD.

#### 3.1.2. Consumption and Degradation of Saccharides and Mannitol

The levels of relevant saccharides and mannitol were determined every 6 h over the total fermentation time of 48 h and the obtained results are presented in Table 3. At 0 h of fermentation, only sucrose (and a small amount of glucose in PRSD) and the raffinose family oligosaccharides verbascose and stachyose/raffinose and galactose were detected in levels above 0.02 %DM for both DFSD and PRSD. The initially available amount of sucrose was with 1.93 %DM only slightly, but significantly, higher in DFSD than in PRSD with 1.42 %DM. The sucrose level rapidly decreased in both substrates and dropped below the LOQ of 0.02 %DM after 6 and 12 h in PRSD and DFSD, respectively. Glucose was only detected in very small quantities just above the LOQ at 6 h of fermentation (and 0 h in PRSD) in both substrates. Fructose was measured in both DFSD and PRSD at 6 and 12 h of fermentation with a decreasing trend. A considerable amount of mannitol, accounting for 0.60 %DM in DFSD and 0.51 %DM in PRSD, was produced within the first six hours of fermentation. The mannitol level then further increased significantly in both substrates reaching its maximum after 12 h in DFSD and after 18 h in PRSD. Thereafter, a relatively stable level of mannitol with a slight decreasing tendency was determined at all timepoints until the maximum fermentation time of 48 h in both substrates. *Leuconostoc* species have been reported to convert a majority of sucrose into glucans (e.g., dextrans) and fructose via glucansucrases [30]. However, especially at early stages of fermentation, sucrose is primarily used as carbon source to support growth. High glucansucrase activity and high glucan yields are mainly observed subsequent to maximum growth rates (during stationary growth phase), for fermentations with sucrose supplementation and where cells were grown in sucrose containing media during inoculum preparation [30,49,50,51]. For example, Xu et al. [26,27,28] and Wang et al. [25] reported substantial glucan production when fermenting sucrose supplemented faba bean flour with several *Leuconostoc* and *Weisella* strains (including the *Ln. citreum* strain DSM 5577) which were mostly grown in sucrose containing media prior to inoculation. The inoculums in the present study were prepared in MRS broth containing glucose as the main carbon source and little sucrose was available in both substrates. Therefore, it can be assumed that the majority of sucrose available in PRSD and DFSD was absorbed by the TR116 cells and subsequently converted into glucose-1-phosphate and fructose by sucrose phosphorylase utilising glucose as carbon source [33,52]. This is further in line with the quick increase of lactic and acetic acid levels (see Table 2), which are, besides ethanol, the main metabolites of glucose catabolism. While the proportions of the main metabolites generally depend on growth conditions, FQ values below 1, as observed in the present study, are mostly unusual in sourdough-like fermentations and lactic acid is commonly produced in molar excess of acetic acid. It has been reported, that under aerobic or semi-aerobic conditions and in the presence of alternative electron acceptors (other than O${}_{2}$), the conversion of acetyl-P into acetate is preferred (additional gain of ATP) over its reduction to ethanol and simultaneous regeneration of NADH to NAD ${}^{+}$ [30,33,51]. This, potentially in combination with a partial incorporation of lactic acid in peptidoglycans, can lead to a substantial decrease in the molar ratio between lactic and acetic acid [30,51,53]. Several *Leuconostoc* species, and *Ln. citreum* TR116 in particular, have been reported to utilise fructose as an alternative electron acceptor to regenerate NAD ${}^{+}$ from NADH by reducing (mannitol-dehydrogenase) it to mannitol [30,31,32,33,35]. This is in agreement with the determined fructose and mannitol concentrations in DFSD and PRSD in the present study which simultaneously decrease (fructose) and increase (mannitol). The values indicate that the majority of fructose released from sucrose (considering the sucrose level in DFSD and PRSD at 0 h) was converted into mannitol. In agreement with the significantly higher sucrose level at 0 h in DFSD than in PRSD, DFSD exhibits higher concentrations of produced mannitol. Xu et al. [26,27] also observed substantial mannitol production in combination with FQs as low as 0.77–0.9 for *Leuconostoc pseudomesenteroides* and *Leuconostoc citreum* strains when they performed single-strain fermentations of faba bean flour. However, low FQs primarily occurred when faba bean flour was supplemented with sucrose for fermentation. The impact of the conversion of fructose released from sucrose into mannitol on the organic acid profile (increase of acetate) has also been reported by Erten et al. [54], Wisselink et al. [55] and Galle et al. [56] for several *Leuconostoc* and *Weissella* strains and by Rice et al. [33] for the *Ln. citreum* strain TR116 used in the present study.

In addition to the carbohydrates discussed above, the levels of the α-galactosides verbascose and stachyose/raffinose as well as galactose were determined in this study (Table 3). α-Galactosides, also known as galactooligosaccharides or raffinose family oligosaccharides (RFO), are plant storage carbohydrates (particularly abundant in the seeds of legumes) consisting of α(1-6) linked galactose molecules (one for raffinose, two for stachyose and three for verbascose) which are bound to sucrose. These compounds are not degraded during human gastro-intestinal digestion due to the absence of α-galactosidase and have therefore been associated with both antinutritional (causing flatulence) as well as prebiotic characteristics [57,58]. In foods containing high levels of RFOs, a moderate reduction of these compounds seems generally desirable from a nutritional perspective. Similar concentrations of the saccharides were determined in DFSD and PRSD at 0 h. Verbascose and stachyose/raffinose were present in considerable amounts accounting for 0.90 %DM and 0.96 %DM (DFSD) and 0.84 %DM and 0.95 %DM (PRSD), respectively. Galactose, on the other hand, was detected in much lower quantities with 0.04 %DM for DFSD and 0.13 %DM for PRSD. Throughout the fermentation process, a generally decreasing trend was observed for verbascose and stachyose/raffinose in both substrates, while galactose accumulated. The profile of stachyose/raffinose concentrations is characterised by an initial plateau (DFSD) or slight increase (PRSD) followed by a steady decrease starting at 18 h of fermentation in DFSD and between 24 and 30 h of fermentation in PRSD. This first accumulation of stachyose/raffinose is expected due to the equal abundance of verbascose and stachyose/raffinose and the fact that only a small proportion (approximately one-sixth) of the initially detected stachyose/raffinose was identified as raffinose (additional separation on HPLC column, data not shown), which is in agreement with the findings of Xu et al. [26,27,28]. Therefore, the degradation of verbascose as well as stachyose to the respective breakdown products stachyose and raffinose causes an initial plateau/increase of the sum value for stachyose and raffinose. The observed degradation of RFOs is likely related to α-galactosidase activity during fermentation. Whether this α-galactosidase activity originates from endogenous α-galactosidase present in faba bean raw material [59] or from microbial α-galactosidase [60,61,62] expressed by *Ln. citreum* TR116 cannot be determined based on these results. However, the inability of *Leuconostoc citreum* to utilise RFOs due to the lack of α-galactosidase has been mentioned in the literature as one of the distinguishing traits in comparison to other *Leuconostoc* species such as *Leuconostoc mesenteroides* [63,64]. Therefore, a breakdown by endogenous faba bean α-galactosidase seems more likely. This is also in line with the findings of Xu et al. [26] who showed a substantial degradation (extant comparable to the present study) of RFOs as well as accumulation of galactose when they used the *Leuconostoc citreum* strain DSM 5577 to ferment native faba bean flour. Moreover, they observed no RFO degradation in a faba bean flour which was autoclaved prior to fermentation to inactivate plant-derived α-galactosidase. Considering the chemical composition of RFOs and the substantial accumulation of galactose in DFSD and PRSD in the present study, it can additionally be assumed that the RFO degradation led to the release of sucrose. However, as the decrease of RFO levels is quite slow, this sucrose could have been available only at later stages of fermentation when the growth of *Ln. citreum* had reached its stationary phase and in small quantities. Interestingly, no fructose accumulation and no further increase in mannitol was observed which indicates that fructose might have been utilised differently than at early stages of fermentation during exponential growth phase. As the main metabolites of carbohydrate catabolism lactic and acetic acid still show a steady, but slower, increase after the first 18 to 24 h (see Table 2), both fructose and glucose released from sucrose were potentially used as carbon source. However, also a utilisation of glucose for the production of small amounts of glucans via glucansucrases as observed by Xu et al. [26] for the fermentation of faba bean flour by *Ln. citreum* DSM 5577 cannot be excluded. Furthermore, the continuously low FQs (high acetate production relative to lactate) in combination with the lack of additional mannitol production indicates the presence of other alternative electron acceptors besides fructose. The observed accumulation of galactose is in agreement with the literature where the inability of *Leuconostoc citreum* strains to utilise galactose has repeatedly been reported [26,63,64]. However, considering the theoretical amount of galactose released from RFOs (mass balance), the quantity of accumulated galactose appears to be unexpectedly high and the origin of a proportion of galactose remains unexplained. It has been reported that faba beans contain considerable amounts of arabinogalactans and arabinogalactan-peptides, which belong to a class of compounds known as proteoglycans [65]. Due to proteolytic activity and acidification during the fermentation process, further galactose might have been released from these compounds.

#### 3.1.3. Contents of Antifungal Phenolic Acids

Phenolic compounds, such as phenolic acids, are known to exert various biological activities. In plant-based food matrices, like faba bean flours, they often occur esterified or bound to proteins or carbohydrates and only a smaller proportion is present in a free form as acids [66,67,68]. Phenolic compounds have also been shown to be metabolites of LAB and to be present in various fermented food products [69,70,71]. Besides their potential antioxidant activity in vitro and in vivo after ingestion and related health benefits [66,72,73,74], phenolic acids have been reported to be advantageous with regard to the microbial shelf life of food products [75,76,77,78,79]. Specifically mould growth, which is one of the main causes of spoilage for bakery products [80], was found to be delayed in sourdough breads when high amounts of phenolic acids were produced/released during sourdough fermentation [44,81,82]. This positive impact on mould spoilage was attributed to a synergistic effect of the reduced pH, produced organic acids like lactic and acetic acid and additionally present phenolic acids [70,71,78,81,83]. In the present study, DFSD and PRSD were screened for the presence of 15 phenolic compounds/acids with antifungal activity. Only the five compounds presented in Table 4 were detected in quantities exceeding the LOQ of 2.50 mg/kg DM.

4-Hydroxybenzoic acid was found in both DFSD and PRSD at all fermentation time points. The levels are relatively stable throughout the fermentation. However, a slight increasing tendency was observed in DFSD; the corresponding difference between 0 h and 48 h levels thereby accounts for +29%. Caffeic acid, coumaric acid and ferulic acid, which are hydroxycinnamic acid derivatives with similar chemical structures, were also found in the fermentates in this study. While ferulic acid could be quantified in DFSD and PRSD at 0 h, caffeic and coumaric acid were not present in levels above the LOQ at the start of the fermentation process. For all three hydroxycinnamic acid derivatives, a clear increase in concentrations was observed during fermentation reaching their highest levels at 42 h or 48 h of fermentation (except for coumaric acid in DFSD, where continuously low levels between 2.52 mg/kg DM (30 h) and 2.99 mg/kg DM (48 h) were measured). The determined increase in ferulic acid was similar for PRSD and DFSD with +72% and +63%, respectively. Caffeic acid and coumaric acid were present in levels exceeding the LOQ at shorter fermentation times in PRSD (6 h and 18 h, respectively) than in DFSD (12 h and 30 h, respectively). Additionally, significantly higher contents of caffeic and coumaric acid were reached after 48 hours of fermentation in PRSD (9.06 mg/kg DM and 6.27 mg/kg DM, respectively) than in DFSD (6.45 mg/kg DM and 2.99 mg/kg DM, respectively). The highest concentrations at the maximum fermentation time of 48 hours were observed for phenyllactic acid accounting for 29.40 mg/kg DM in DFSD and 33.43 mg/kg DM in PRSD. This represents a drastic increase, as phenyllactic acid was below 2.50 mg/kg DM at 0 h of fermentation and started exceeding the LOQ only at 12 h of fermentation in DFSD and at 6 h in PRSD. While *Ln. citreum* TR116 has not previously been associated with the production of phenolic acids, the observed increase in phenolic acids contents during fermentation of DFSD and PRSD could be related to the metabolic pathways of this strain. Specifically, phenyllactic acid, which is the phenolic acid with the highest increase in this study, has been described as a metabolite of LAB and was found to be produced by transamination and subsequent reduction of phenylalanine [84,85]. The fact that FB protein exhibits a relatively high abundance of phenylalanine and that both DF and PR are relatively rich in protein theoretically leads to a high availability of this precursor for phenyllactic acid in both substrates [18]. This is further supported by the tendency towards slightly higher phenyllactic acid levels reached in PRSD. However, also the possibility of a release of plant-derived phenolic acids due to a degradation of plant constituents the phenolic acids are usually bound to (proteins and carbohydrates) has been mentioned in the literature and seems to be a probable explanation for the trends observed in this study [86,87,88,89]. Especially the hydroxycinnamic acid derivatives might be released rather than produced during fermentation, as these compounds have been reported to occur largely as ester conjugates in faba bean plants [90,91,92,93,94,95]. The release can be mediated by proteolytic activity and acidification during fermentation or by activity of endogeneous or microbial enzymes. Feruloyl esterases, for example, have been found to be produced by various LAB species and to degrade the ester bonds of hydroxycinnamoyl esters [75,86,96]. The more pronounced increase of the three hydroxycinnamic acid derivatives in PRSD suggests that air classification might lead to a higher abundance of bound phenolic acids in the high-protein fraction (PR). The findings in the present study are in agreement with Coda et al. [97] who reported an increase in total phenolic content and antioxidant activity during sourdough-type propagation of a dehulled FB flour and an air-classified high-protein FB flour. They also associated this trend with improved extractability of phenolics due to lactic acidification and potential esterase activity. As mentioned above, the continuously low FQs (high acetate production relative to lactate) in combination with the lack of additional mannitol production at later stages of fermentation (from fructose liberated from RFOs) indicate the presence of other alternative electron acceptors besides fructose. It has been described that phenolic compounds such as hydroxycinnamic acids can be used as alternative electron acceptors by LAB [69,86,98]. With regard to a beneficial effect of the increased content of phenolic acids both their antioxidant and antifungal activity should be considered. Although a low pH was described to be part of the synergistic antifungal effect in fermented foods and the pH drop observed for DFSD and PRSD in this study was quite moderate, a positive influence on the microbial shelf life of breads containing DFSD and PRSD can be expected.

### 3.2. Baking Properties of Sourdough-Like Fermentates

To evaluate the baking performance of the produced FB fermentates, five different bread formulations were prepared. In addition to a wheat flour control, FB breads were produced by replacing 15% of wheat flour by either unfermented or fermented (48 h) FB ingredient (based on solids in ingredient). For the FB ingredients PR (unfermented) and PRSD (fermented), this replacement level ensures a protein content of >20% of calories provided by protein in the final bread products (calculated based on compositional data of ingredients and recipe according to Table 1). This level is defined as the requirement for a “high in protein” nutrition claim made on food products according to European legislation [99].

#### 3.2.1. Water Absorption and Gluten Aggregation

The influence of the partial replacement of wheat flour by unfermented and fermented FB ingredients on water absorption and gluten aggregation characteristics was determined by Farinograph and GlutoPeak measurements. The water amount required to reach a dough consistency of 500 FU in the Farinograph measurements was determined for the five respective (theoretical) flour mixtures and used as optimal water addition level for the baking trials. The determined Farinograph water absorptions (FWAs) also reflect the impact of FB ingredient incorporation and FB ingredient fermentation on the water absorption characteristics in wheat flour based bread dough systems. The obtained FWAs are displayed in Table 5. Although significant differences were detected, all of the FWAs determined in this study were relatively similar considering the big differences which can be observed just between different wheat flours [100]. The replacement of 15% wheat flour led to no change in FWA for DF flour mix and only a small decrease was observed for PR flour mix. The reduced FWA for PR flour mix might be associated with the higher protein content and the relatively high solubility of faba bean protein as described by Hoehnel et al. [11]. The incorporation of the fermented FB ingredients resulted in a significant increase in FWAs in comparison to the respective flour mixtures with unfermented FB ingredients as well as in comparison to wheat flour alone. Organic acids are known to increase FWAs [101,102]. A higher number of charges (like dissociated acids) in the system increases intramolecular repulsive forces and therefore promotes the unfolding of gluten proteins which leads to more swelling and higher water absorption [103]. Furthermore, Vogelsang-O’Dwyer et al. reported a drastically decreased solubility of faba bean protein between pH 3.6 and 6.0 [18]. Both FB fermentates in the present study exhibit pH values in this range. This can lead to an increased water absorption of the faba bean proteins in DFSD and PRSD compared to DF and PR, respectively. The fact that the FWA increase was more pronounced for PRSD flour mix, which contains more faba bean protein, than for DFSD flour mix further supports this hypothesis.

**Figure 2:**
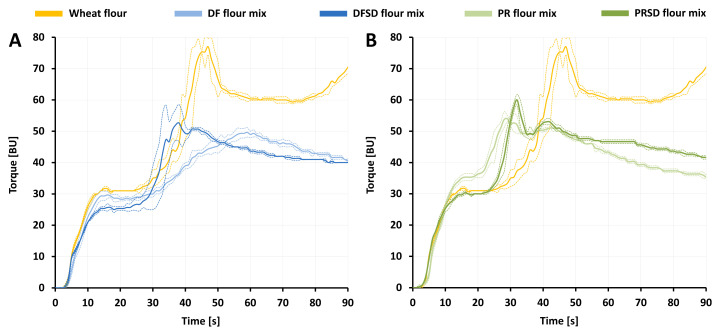
Flour properties of wheat flour and HP flour mix: (Gluten-aggregation profiles obtained by GlutoPeak test: (**A**) DF flour mix, DFSD flour mix compared to wheat flour; (**B**) PR flour mix and PRSD flour mix compared to wheat flour. Dashed curves represent standard deviation.

The gluten aggregation properties of wheat flour and flour mixtures containing FB ingredients were determined using the GlutoPeak device. The measurements indicated striking differences between the five samples and the variables obtained from the curves are presented in Table 5. A PMT of 45.7 s and a TM of 77.0 BU was measured for wheat flour. The partial replacement of wheat flour by DF and PR led to significantly lower TMs accounting for 49.7 BU and 54.7 BU, respectively. While the TM was reached significantly earlier for PR flour mix (32.0 s), a delay was observed for DF flour mix (58.5 s). The values obtained for DFSD flour mix and PRSD flour mix suggest that fermentation leads to a more similar impact of the two ingredients on gluten aggregation as opposed to the differing influences found for DF flour mix and PR flour. No significant difference was detected for the TMs of DFSD flour mix and PRSD flour mix with 56.7 BU and 58.3 BU, respectively. Furthermore, the TMs were reached significantly earlier than for wheat flour in both DFSD flour mix (36.7 s) and PRSD flour mix (32.0 s). A general trend with earlier and higher peaks for stronger wheat flours with higher contents and quality of gluten has been reported in the literature for measurements of pure wheat flours [104,105,106]. Due to the partial replacement of wheat flour, all FB flour mixtures contain considerably less gluten, and generally lower as well as later gluten peaks could have been expected for these samples. Additionally, based on this general trend, the values indicate improved gluten aggregation in DFSD flour mix (significantly lower PMT and higher TM) and PRSD flour mix (significantly higher TM) when compared to DF flour mix and PR flour mix, respectively. However, it has been shown that more factors than solely the gluten content affect GlutoPeak measurements [11,12,35,107,108] and, as previously suggested by Hoehnel et al. [11], partial wheat flour replacement leads to complex changes in gluten aggregation profiles which do not necessarily follow this general trend. This is why, in addition to PMTs and TMs, a comparative evaluation of the gluten aggregation profiles (Figure 2) is required.

The GlutoPeak curve of wheat flour shows a well-described profile of initial torque increase, equilibrium plateau, rapid torque increase and gluten peak followed by a decrease in torque caused by a beginning breakdown of the gluten network. Goldstein et al. [109] associated a fast gluten network development (fast torque increase), a sharp gluten peak and rapid breakdown with weak flours. The more pronounced equilibrium plateau in DF flour mix, DFSD flour mix and PRSD flour mix therefore suggests the formation of a stronger gluten network when compared to PR flour mix. Bouachra et al. [107] described a drastically accelerated gluten aggregation in the presence of lactic acid, which is in agreement with the difference in PMT between DF flour mix and DFSD flour mix. However, Bouachra et al. [107] also associated this trend with low pHs caused by the addition of lactic acid and a related increase in positive charges in the system which increases intramolecular repulsive forces, unfolding of gluten proteins and their aggregation. The fact that there was no significant change in PMT between PR flour mix and PRSD flour mix could be related to the considerably less pronounced pH drop during fermentation of PRSD compared to DFSD. Another remarkable change in the gluten aggregation profiles is the more distinct second peak in DFSD flour mix and PRSD flour mix when compared to the respective flour mixtures containing unfermented FB ingredients. This second peak has previously been attributed to potential co-networking of gluten and non-wheat proteins subsequent to a beginning breakdown of the gluten network [11]. The presented results therefore suggest a stronger co-networking of faba bean proteins subjected to the fermentation process with gluten. The slightly more acidic pH faba bean proteins were subjected to during fermentation might have altered their structure and led to partial unfolding and an associated exposure of hydrophobic regions which are shielded within the native protein structure at a neutral pH. This could, in turn, facilitate co-networking with gluten proteins as hydrophobic interactions play an important role during gluten network formation. Overall, the GlutoPeak measurements show inferior gluten aggregation in flour mixtures containing FB ingredients in comparison to plain wheat flour. However, also a substantial improvement of gluten aggregation behaviour of flour mixtures containing FB ingredients induced by the fermentation process was indicated.

#### 3.2.2. Bread Quality

**Figure 3:**
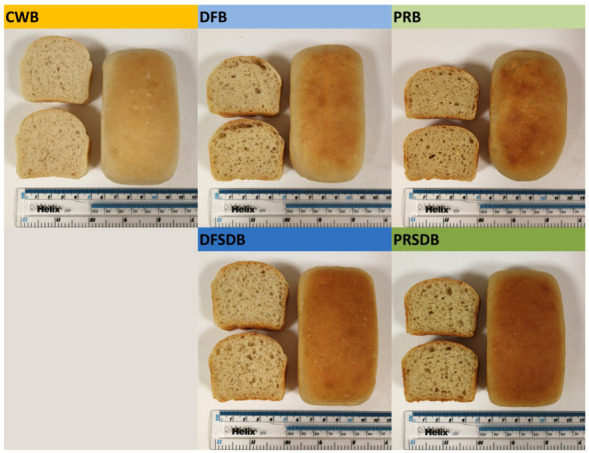
Photographs of control wheat bread and faba bean breads.

Photographs of the five bread formulations produced in this study are displayed in Figure 3.

A visual evaluation of the photographs immediately reveals striking differences in size, crumb structure, crust colour and crumb colour of these bread formulations. The determined technological bread quality characteristics confirm this observation and are presented in Table 6.

Only small differences in dough pH were observed. The incorporation of unfermented FB ingredients DF and PR led to no significant changes in dough pH. Even the slightly lower pH of the PRSDB dough did not represent a significant difference compared to CWB. Only the incorporation of DFSD caused a significantly lower pH of the bread dough, which is in line with the more pronounced pH drop observed for DFSD than for PRSD. The bake loss of DFB (11.4%) and PRB (10.6%) was significantly lower than that of CWB (12.0%). This trend was not observed for the breads containing the fermented FB ingredients which showed bake losses higher than or similar to CWB with 12.8% (DFSDB) and 12.2% (PRSDB). With regard to one of the most indicative bread quality characteristics, the specific volume (SV), the incorporation of all FB ingredients (DF, DFSD, PR and PRSD) led to a significant decrease when compared to CWB with an SV of 3.09 mL/g. However, the extent of this general trend differed among the FB ingredient containing bread formulations. The incorporation of the unfermented ingredient PR led to the lowest SV observed in this study (2.16 mL/g). For DF, only a moderate decrease in SV (to 2.42 mL/g) was found. This suggests a generally better baking performance of DF when compared to PR. Interestingly, fermentation seems to significantly improve the baking performance of both FB ingredients. The extent to which fermentation improved the FB ingredients’ baking performance was similar. DFSDB showed with 2.96 mL/g an SV, which was significantly higher than that of DFB and only slightly lower than that of CWB. Morever, PRSDB had with 2.58 mL/g a much higher SV than PRB and even reached a higher SV than DFB. This agrees well with the results obtained from GlutoPeak measurements which indicated a better gluten aggregation behaviour with potentially improved gluten quality in the presence of both fermented FB ingredients as opposed to unfermented DF and PR. Crumb hardness is another important indicator of bread quality. A strong negative correlation of crumb hardness determined after 0 days of storage with SV was observed in this study (r:−0.99). Hardness values (0 d of storage) therefore follow the same trends (inverse) as described for SVs. The significantly lower crumb hardness of DFSDB and PRSDB (compared to DFB and PRB, respectively) represents further evidence that the performed sourdough-like fermentation of DF and PR leads to a substantial upgrade of the ingredients’ baking performance. In addition, crumb hardness was determined after two and five days of storage. The calculated staling rates show that breads containing FB ingredients have a smaller increase in crumb hardness during storage, relative to their initial crumb hardness. However, also DFB, DFSDB, PRB and PRSDB still show substantial staling and, compared to CWB, significantly harder crumb after two and five days of storage. With regard to the bread formulations’ crumb structure the variables number of cells and cell area were evaluated. The number of cells significantly decreased when FB ingredients were included in the formulation (except for PRB, number of cells with 1454 not significantly different from that of CWB with 1527). This decrease was significantly more pronounced when the fermented instead of unfermented FB ingredients were applied. Furthermore, a reduced cell area was measured for DFB (46.9%) and PRB (46.3%) in comparison to CWB with 48.4%. The breads containing DFSD and PRSD on the other hand showed with 49.6% and 49.4% cell areas similar to CWB. These results indicate a denser crumb structure in DFB and PRB, while DFSDB and PRSDB exhibit crumb structures more similar to CWB with a slight tendency towards bigger cells, which can also be visually observed in the photographs presented in Figure 3. This, in addition to the higher SV, might have contributed to the lower crumb hardness values determined for DFSD and PRSD in comparison to DFB and PRB, respectively. Both crust and crumb lightness were reduced due to application of FB ingredients. Considering the fact that both raw materials PR and DF exhibits lower lightness values than wheat flour (data not shown), such a trend was expected. Moreover, Hoehnel et al. [11] reported a substantially reduced lightness of crust when 15% of wheat flour was replaced by PR. Additionally, the decrease in lightness of crust and crumb was stronger for breads containing DFSD and PRSD. This can be associated with a potential proteolytic activity during fermentation (either by activated plant-derived enzymes due to pH drop or mediated by microbial proteolytic enzymes produced by TR116). This is likely to increase the availability of components (mainly primary amines) involved in the Maillard reaction, which is besides caramelisation the main contributor to crust browning [110,111]. Several previous studies investigating faba bean fermentation largely focused on EPS (dextrans) production as a potential tool to improve the techno-functional value of faba bean flours including its rheological properties and baking performance [25,27,112]. Wang et al. [25] prepared sourdough-like fermentates from faba bean flours with two different EPS producing LAB strains (*Weissella confusa* VTT E-143403 and *Leuconostoc pseudomesenteroides* DSM 20193) with and without sucrose supplementation to trigger EPS production. Breads were prepared by replacing 30% of wheat flour by faba bean fermentates (based on solids). While faba bean fermentates produced with the *Weissella* strain (with and without sucrose supplementation) significantly improved bread volume, a negative impact was observed for the *Leuconostoc* fermentate (+ sucrose). This difference was associated with a lower amount of dextrans produced by the *Leuconostoc* strain, a potentially different, and less beneficial, polymer structure of the dextrans as well as a higher acidity of the *Leuconostoc* fermentates (as intensive acidification was shown to negatively affect bread volume, crumb hardness and staling [113]). Also Coda et al. [114] observed a decreased bread volume and increased crumb hardness (in comparison to both a wheat flour control and composite faba bean/wheat bread) when 30% of wheat flour were replaced by a faba bean ingredient fermented with *P. pentosaceus* I02. In the present study, no major EPS production can be expected as explained in the sections concerning the fermentation profile of *Ln. citreum* TR116 in DFSD and PRSD above. However, a substantial improvement of the baking performance of both FB ingredients was achieved through fermentation. GlutoPeak results in combination with the fermentate characterisation and the obtained bread quality characteristics reveal that the improvement of the FB ingredients’ baking performance is largely related to an improved gluten aggregation in the presence of fermented FB ingredients as opposed to unfermented DF and PR.

### 3.3. In Vitro Starch Digestibility and Indication of Glycaemic Index

**Figure 4:**
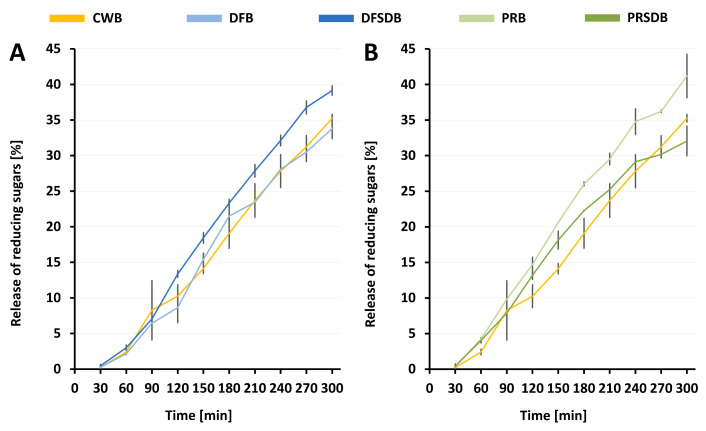
Sugar release curves expressed as reducing sugars as percentage of digestible starch: (**A**) DFB and DFSDB compared to CWB; (**B**) PRB and PRSDB compared to CWB.

Besides technological characteristics, the nutritional quality of bread is crucial. The kinetics of starch digestibility in vivo are of major importance and indicative of the resulting blood sugar levels and, therefore, the glycaemic index (GI) of foods. An elevated risk for certain types of cancer, heart disease and diabetes has been associated with the consumption of high-GI and high-GL diets that are primarily based on food products containing high levels of rapidly digestible starch [115,116,117,118,119]. It has been reported that the food structure can have a major impact on the accessibility of starch by pancreatic α-amylase and, therefore, on starch digestibility [120,121,122]. In pasta, for example, it is believed that the dense structure and coagulated protein network surrounding the starch granules, which is formed during the cooking process, delays starch accessibility by enzymes, leading to a low GI [123,124]. Furthermore, Holt et al. [125] observed a lower glycaemic response for a high-protein bread when equal-energy portions of regular wheat bread and a high-protein bread were consumed. But also dietary fibre compounds, and specifically those that cause an increase in viscosity, can lead to a delay in starch digestibility [121]. The performance of a starch digestion in vitro and the evaluation of obtained sugar release curves can give an indication of sugar release and blood glucose response in vivo. The curves for the release of reducing sugars (expressed as a percentage of digestible starch) for all five bread samples investigated in the present study are displayed in Figure 4.

The curves for all the five bread formulations appear quite similar at first sight, which indicates that their release of reducing sugars during amylolytic treatment is similar despite varying contents of digestible starch. A closer look reveals several details which distinguish breads containing fermented FB ingredients from those that contain unfermented FB ingredients. Furthermore, a difference between DFB and DFSDB on one side and PRB and PRSDB on the other side can be observed. While DFB and DFSDB both exhibit similar sugar release as CWB within the first 90 min, further treatment with α-amylase thereafter leads to a gap between DFB and DFSDB. DFB closely follows the curve of CWB, whereas the starch in DFSDB seems to be more susceptible to a rapid breakdown. Interestingly, an assessment of the curves obtained for PRB and PRSDB reveals a trend opposing that of DFB and DFSDB. Here, the starch in the bread containing the unfermented ingredient (PRB) appears to be rapidly digested and PRSDB shows the flatter curve, more similar to that of CWB. However, the PRSDB curve still indicates a slightly faster starch digestion than for DFB. Furthermore, the initial release of reduced sugars within the first 150 min of in vitro digestion is generally higher for PRB and PRSDB than for DFB and DFSDB. These trends are also well reflected by the calculated hydrolysis indices (HIs), which are presented in Table 7.

**Figure 5:**
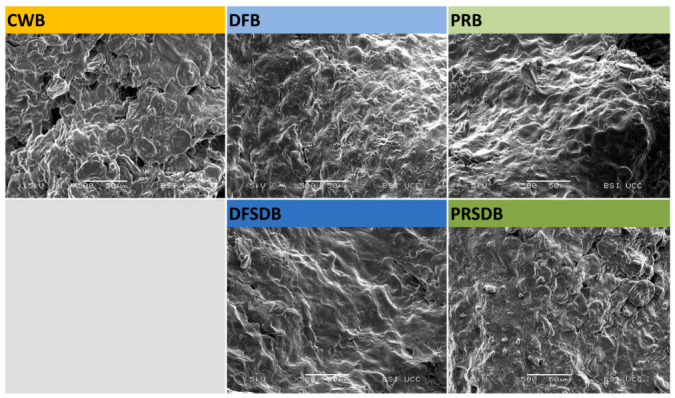
Micrographs (obtained by SEM) of crumb from control wheat bread and faba bean breads.

DFB exhibits a HI of 99.9% which indicates a sugar release within 240 min of digestion that is very similar to CWB. Fermentation of DF led to an accelerated starch digestion represented by a HI of 119.0% determined for DFSDB. The highest HI in this study was obtained for PRB with 133.4%. For this FB ingredient, the HI of PRSDB (114.9%) indicates that fermentation caused a slower starch digestion. The fact that, for DF, fermentation seemed to accelerate or facilitate starch digestion in the present study does not follow the trends observed by Coda et al. [22] who performed in vitro starch digestion trials with both fermented faba bean flour as well as fermented air-classified faba bean high-protein flour. They found significantly decreased HIs in both fermented faba bean ingredients compared to the unfermented flours. However, these results were determined for the raw ingredients. Therefore, they do not account for the impact of FB ingredients on starch originating from wheat flour or for the influence of processing conditions during bread production on starch digestibility. Still, in another study, Coda et al. [114] reported a decreased GI when they replaced 30% of wheat flour by faba bean sourdough (based on solids). Altough Gobbetti et al. [76] mentioned a generally reducing effect of the use of sourdough technology on the glycaemic response of bread products, it was pointed out that varying results were reported in the literature depending on processing conditions, final bread quality and the application of dietary fibre or wholegrain ingredients. A decrease in GI in connection with sourdough fermentation was also associated with the presence of low pH values between 3.5 and 4.0 [76]. Furthermore, lactic acid was shown to be one of the main factors to lower the rate of starch digestion in in vitro trials [76,121,126]. Acetic acid and propionic acid on the other hand were found to play a role in reducing the rate of gastric emptying [76,121,126,127] which is an effect that cannot be accounted for with the in vitro digestion method performed in the present study. Scazzina et al. [128] concluded that the mechanism through which sourdough fermentation reduces the GI of breads does not seem to be related to an alteration of the rate of starch hydrolysis. Another key factor for starch digestibility is its gelatinisation. A higher degree of gelatinisation generally correlates with higher rates of starch hydrolysis as the amorphus structure of gelatinised starch offers an increased availability of binding sites for α-amylase [129,130]. The results of the present study do not seem to agree with these generally observed trends reported in the literature. However, the presented findings can potentially be explained considering the major differences between the applied FB ingredients. The primary constituent of DF is starch. PR on the other hand contains mainly protein and only a very small amount of starch. While the incorporation of DF did not seem to affect the rate of starch hydrolysis, the fermented version of DF, DFSD, led to an increased susceptibility of starch in DFSDB to hydrolysis. It can be hypothesised that the fermentation process made faba bean starch more accessible, more susceptible to gelatinisation during baking and, therefore, more rapidly digestible. In the case of PRB and PRSDB, however, this cannot be a valid explanation because of the very low starch content of PR. Water addition levels for the bread formulations in this study were determined in Farinograph trials. While faba bean protein is likely to contribute to this water absorption (late peak visible in farinograms, data not shown) it might not hold water as well as for example gluten. Gluten is known to have thermosetting properties, which leads to a moderate water redistribution during baking and facilitated starch gelatinisation. In the presence of faba bean protein, more water might be available for starch gelatinisation and a higher degree of gelatinisation could be the result. Higher water availability might be caused by a lower water holding capacity of faba bean proteins when heat treated or by the weakened gluten network in the presence of faba bean proteins as observed in GlutoPeak measurements. This would explain the substantially increased HI determined for PRB in the present study. It has been described above that fermentation and the associated moderate reduction of pH might induce changes in the faba bean protein structure (unfolding, agglomeration) in PRSD which is in agreement with the increased FWA and potentially improved co-networking with gluten proteins indicated by the GlutoPeak curve. These structural changes and the improved gluten aggregation might lead to lower water availability for starch gelatinisation during baking when compared to PRB and could be an explanation for the lower HI of PRSDB in comparison to PRB. This also means that, specifically for PRB and PRSDB, an adjustment of the processing conditions during bread production, such as water addition level or mixing time, could be used as a tool to reduce rapid starch hydrolysis. The explanations given are also in agreement with the micrographs obtained from the crumbs of all five bread formulations in this study (Figure 5). The crumb of breads containing FB ingredients generally appears to have a smoother structure with starch granules being less visible than in CWB. This trend seems to be more pronounced in PRB and DFSDB, while for DFB and PRSDB individual exposed starch granules (like in CWB) can still be observed. This could indicate a higher degree of starch gelatinisation in PRB and DFSDB and explain the high HIs determined for these samples.

The presented results show a similar or slightly more rapid/increased sugar release during in vitro starch digestion of breads containing FB ingredients in comparison to CWB. This could indicate slightly higher GIs, especially of DFSDB, PRB and PRSDB. However, as mentioned above, the GI reducing effect of sourdough fermentation is not necessarily related to the rate of starch hydrolysis. Due to the relatively high levels of acetic acid in both DFSD and PRSD, a substantial delay of gastric emptying might be observed in vivo and therefore improved glycaemic responses in comparison to CWB.

## 4. Conclusions

This study compares the fermentation characteristics of the multifunctional *Ln. citreum* strain TR116 in two faba bean (FB) ingredients: faba bean dehulled flour (DF) and air-classified faba bean high-protein flour (PR). With regard to microbial growth, carbohydrate consumption as well as production of lactic and acetic acid, TR116 showed a similar metabolism in both substrates. The most important differences include a much more moderate pH drop, a slightly more pronounced galactose accumulation and a higher content of phenolic acids after 48 h of fermentation for PRSD in comparison to DFSD. Fermentation with *Ln. citreum* TR116 caused a striking improvement of the ingredients’ baking performance when DFSD and PRSD were compared to their unfermented counterparts (including increased specific volume of bread loaves and decreased crumb hardness). GlutoPeak measurements allowed for an association of this observation with an improved gluten aggregation in the presence of fermented FB ingredients. The high potential of the produced faba bean fermentates as functional ingredients for bakery applications is further underlined by the presence of phenolic acids with antifungal activity which might improve the microbiological shelf life of DFSD or PRSD containing breads. Furthermore, the accumulation of small amounts of mannitol could positively influence sensory characteristics of the final bread products when compared to breads containing unfermented FB ingredients. In general, PRSD seems to represent a better choice than DFSD as a functional ingredient in bakery products. It offers a higher potential to improve protein quality in cereal products at lower levels of wheat flour replacement due to its higher protein content. This higher protein content also secures the opportunity to label breads in which 15% of wheat flour are replaced by PRSD with a “high in protein” claim according to European legislation [99]. PRSD further offers better technological bread quality than both PR and DF as well as slightly higher levels of acetic acid and phenolic acids than DFSD, which is related to a higher potential to show improved microbial shelf life when incorporated in bakery products. In addition to this, HIs of DFSDB (119.0%) and PRSDB (114.9%) were similar, but a small tendency towards a lower HI of PRSDB was observed. While this might still leave DFSD in favour as a functional bakery ingredient, also the generally lower content of digestible starch should be considered (67.06 %DM (DFSDB) and 61.96 %DM (PRSDB)). This can be expected to result in a lower GL of breads containing PRSD (as opposed to breads containing DFSD). Furthermore, the explanations for the increased HI of PRSDB (when compared to PRB and CWB) offered above, promise that a slight adjustment of processing conditions (water level, mixing time and speed) might further reduce starch digestibility. This could have an additional positive impact on quality characteristics of bread formulations containing PRSD.

## Figures and Tables

**Table 1:** Recipes for control and faba bean formulations.

Ingredient	% Based on Flour	% Based on Recipe
Wheat flour	100.0 (85.0 ${}^{†}$)	60.46
Faba bean ingredient (DF/PR/DFSD/PRSD)	0.0 (15.00 ${}^{†,}$${}^{‡}$)	0.00
Baker’s yeast	2.0	1.21
NaCl	2.0	1.21
Oil	1.0	0.60
Water	60.4 (FWA ${}^{†,}$*)	36.52
Total	165.4	100.00

${}^{†}$ For faba bean formulations. ${}^{‡}$ For fermentates: addition level was based on solids in fermentate (e.g., due to dough yield of 200, 30% of faba bean fermentates were used; water addition was adjusted accordingly. * FWA, determined Farinograph water absorption.

**Table 2:** Levels of lactic and acetic acid in faba bean fermentates, values expressed in %DM.

	Ferm. Time [h]	Acetic Acid ${}^{S,T}$	Lactic Acid ${}^{S,T}$	Fermentation Quotient ${}^{S}$
Faba bean	0	n.d.	n.d.	-
dehulled	6	0.45 ± 0.01 ${}^{l}$	0.51 ± 0.01 ${}^{h}$	0.76 ± 0.03 ${}^{cd}$
flour	12	0.69 ± 0.00 ${}^{k}$	0.95 ± 0.02 ${}^{g}$	0.91 ± 0.02 ${}^{ab}$
(DFSD)	18	0.75 ± 0.01 ${}^{jk}$	1.13 ± 0.02 ${}^{ef}$	1.00 ± 0.02 ${}^{a}$
	24	0.84 ± 0.01 ${}^{ij}$	1.23 ± 0.02 ${}^{def}$	0.98 ± 0.02 ${}^{a}$
	30	0.91 ± 0.01 ${}^{hi}$	1.25 ± 0.01 ${}^{cdef}$	0.92 ± 0.02 ${}^{ab}$
	36	1.01 ± 0.02 ${}^{gh}$	1.33 ± 0.02 ${}^{abcd}$	0.88 ± 0.03 ${}^{abc}$
	42	1.04 ± 0.03 ${}^{efg}$	1.37 ± 0.04 ${}^{abc}$	0.88 ± 0.06 ${}^{abc}$
	48	1.16 ± 0.10 ${}^{cd}$	1.32 ± 0.13 ${}^{bcd}$	0.77 ± 0.15 ${}^{cd}$
Faba bean	0	n.d.	n.d.	-
high-protein	6	0.56 ± 0.01 ${}^{l}$	0.64 ± 0.02 ${}^{h}$	0.75 ± 0.02 ${}^{cd}$
flour	12	1.02 ± 0.00 ${}^{fg}$	1.13 ± 0.02 ${}^{f}$	0.74 ± 0.02 ${}^{d}$
(PRSD)	18	1.07 ± 0.02 ${}^{defg}$	1.25 ± 0.02 ${}^{cde}$	0.78 ± 0.02 ${}^{cd}$
	24	1.14 ± 0.03 ${}^{cde}$	1.33 ± 0.03 ${}^{bcd}$	0.78 ± 0.04 ${}^{cd}$
	30	1.12 ± 0.05 ${}^{def}$	1.35 ± 0.04 ${}^{abcd}$	0.80 ± 0.06 ${}^{bcd}$
	36	1.24 ± 0.06 ${}^{bc}$	1.41 ± 0.06 ${}^{ab}$	0.76 ± 0.07 ${}^{cd}$
	42	1.29 ± 0.05 ${}^{ab}$	1.45 ± 0.06 ${}^{a}$	0.76 ± 0.07 ${}^{cd}$
	48	1.37 ± 0.14 ${}^{a}$	1.41 ± 0.13 ${}^{ab}$	0.70 ± 0.14 ${}^{d}$

Means ± standard deviation with different lower case letters in the same column are significantly different at *p* < 0.05. not detected or levels below 0.17 %DM. ${}^{S}$ Two-way ANOVA confirmed significant effect (*p* < 0.05) of substrate (DF/PR) on this variable. ${}^{T}$ Two-way ANOVA confirmed significant effect (*p* < 0.05) of fermentation time (0–6 h) on this variable

**Table 3:** Carbohydrate profile of faba bean fermentates, values expressed in %DM.

	Ferm. Time [h]	Sucrose ${}^{S,T}$	Glucose ${}^{S}$	Fructose ${}^{T,S:T}$	Mannitol ${}^{S}$	Verbascose ${}^{S,T,S:T}$	Stachyose/Raffinose ${}^{S,T,S:T}$	Galactose ${}^{S,T,S:T}$
Faba bean	0	1.93 ± 0.05 ${}^{a}$	n.d.	n.d.	n.d.	0.90 ± 0.02 ${}^{a}$	0.96 ± 0.02 ${}^{cde}$	0.04 ± 0.00 ${}^{l}$
dehulled	6	0.03 ± 0.00 ${}^{c}$	0.07 ± 0.01 ${}^{a}$	0.39 ± 0.00 ${}^{a}$	0.60 ± 0.01 ${}^{fgh}$	0.84 ± 0.02 ${}^{b}$	0.99 ± 0.02 ${}^{bcd}$	0.28 ± 0.01 ${}^{j}$
flour	12	n.d.	n.d.	0.03 ± 0.00 ${}^{d}$	0.82 ± 0.02 ${}^{a}$	0.72 ± 0.03 ${}^{c}$	0.99 ± 0.03 ${}^{bcd}$	0.48 ± 0.01 ${}^{i}$
(DFSD)	18	n.d.	n.d.	n.d.	0.82 ± 0.01 ${}^{a}$	0.56 ± 0.02 ^*e*^	0.90 ± 0.03 ${}^{ef}$	0.66 ± 0.02 ${}^{h}$
	24	n.d.	n.d.	n.d.	0.77 ± 0.01 ${}^{ab}$	0.49 ± 0.01 ${}^{f}$	0.84 ± 0.01 ${}^{g}$	0.75 ± 0.01 ${}^{g}$
	30	n.d.	n.d.	n.d.	0.74 ± 0.01 ${}^{abc}$	0.42 ± 0.03 ${}^{g}$	0.77 ± 0.04 ${}^{hi}$	0.86 ± 0.02 ${}^{f}$
	36	n.d.	n.d.	n.d.	0.72 ± 0.03 ${}^{bcd}$	0.37 ± 0.01 ${}^{h}$	0.71 ± .02 ${}^{j}$	0.95 ± 0.01 ^*e*^
	42	n.d.	n.d.	n.d.	0.70 ± 0.02 ${}^{bcde}$	0.32 ± 0.02 ${}^{ij}$	0.63 ± 0.03 ${}^{k}$	1.03 ± 0.03 ${}^{d}$
	48	n.d.	n.d.	n.d.	0.65 ± 0.11 ${}^{defg}$	0.28 ± 0.01 ${}^{j}$	0.59 ± 0.03 ${}^{k}$	1.12 ± 0.02 ${}^{c}$
Faba bean	0	1.42 ± 0.04 ${}^{b}$	0.03 ± 0.00 ${}^{b}$	n.d.	n.d.	0.84 ± 0.04 ${}^{b}$	0.95 ± 0.05 ${}^{cde}$	0.13 ± >0.01 ${}^{k}$
high-protein	6	n.d.	0.02 ± 0.01 ${}^{b}$	0.25 ± 0.03 ${}^{b}$	0.51 ± 0.03 ${}^{h}$	0.81 ± 0.03 ${}^{b}$	1.03 ± 0.04 ${}^{b}$	0.46 ± 0.00 ${}^{i}$
flour	12	n.d.	n.d.	0.17 ± 0.02 ${}^{c}$	0.58 ± 0.02 ${}^{gh}$	0.76 ± 0.02 ${}^{c}$	1.09 ± 0.03 ${}^{a}$	0.69 ± 0.01 ${}^{h}$
(PRSD)	18	n.d.	n.d.	n.d.	0.67 ± 0.02 ${}^{cdef}$	0.63 ± 0.02 ${}^{d}$	1.01 ± 0.02 ${}^{bc}$	0.87 ± 0.03 ${}^{f}$
	24	n.d.	n.d.	n.d.	0.63 ± 0.02 ${}^{efg}$	0.54 ± 0.01 ^*e*^	0.93 ± 0.03 ${}^{def}$	0.99 ± 0.03 ${}^{de}$
	30	n.d.	n.d.	n.d.	0.63 ± 0.04 ${}^{efg}$	0.47 ± 0.03 ${}^{f}$	0.87 ± 0.04 ${}^{fg}$	1.10 ± 0.06 ${}^{c}$
	36	n.d.	n.d.	n.d.	0.61 ± 0.04 ${}^{efg}$	0.42 ± 0.01 ${}^{g}$	0.83 ± 0.01 ${}^{gh}$	1.24 ± 0.02 ${}^{b}$
	42	n.d.	n.d.	n.d.	0.60 ± 0.04 ${}^{fgh}$	0.36 ± 0.01 ${}^{hi}$	0.75 ± 0.01 ${}^{ij}$	1.34 ± 0.03 ${}^{a}$
	48	n.d.	n.d.	n.d.	0.52 ± 0.09 ${}^{h}$	0.33 ± 0.02 ${}^{hi}$	0.73 ± 0.04 ${}^{ij}$	1.38 ± 0.05 ${}^{a}$

Means ± standard deviation with different lower case letters in the same column are significantly different at *p* < 0.05. n.d., not detected or levels below 0.02 %DM. ${}^{S}$ Two-way ANOVA confirmed significant effect (*p* < 0.05) of substrate (DF/PR) on this variable. ${}^{T}$ Two-way ANOVA confirmed significant effect (*p* < 0.05) of fermentation time (0–6 h) on this variable. ${}^{S:T}$ Two-way ANOVA confirmed significant interactive effect (*p* < 0.05) of substrate and fermentation time on this variable.

**Table 4:** Contents of phenolic acids in faba bean fermentates, values expressed in mg/kg DM.

	Ferm. Time [h]	4-Hydroxybenzoic Acid ${}^{T,S:T}$	Caffeic Acid ${}^{S,T,S:T}$	Coumaric Acid ${}^{S,T,S:T}$	Ferulic Acid ${}^{T,S:T}$	Phenyllactic Acid ${}^{T}$
Faba bean	0	3.57 ± 0.05 ${}^{fg}$	n.d.	n.d.	3.03 ± 0.16 ${}^{h}$	n.d.
dehulled	6	3.80 ± 0.10 ${}^{cdef}$	n.d.	n.d.	3.27 ± 0.21 ${}^{fgh}$	n.d.
flour	12	3.60 ± 0.16 ${}^{efg}$	4.80 ± 0.28 ${}^{gh}$	n.d.	3.25 ± 0.18 ${}^{fgh}$	7.57 ± 0.46 ${}^{gh}$
(DFSD)	18	3.87 ± 0.04 ${}^{bcdef}$	5.82 ± 0.21 ${}^{efg}$	n.d.	3.69 ± 0.19 ${}^{defg}$	10.45 ± 0.21 ${}^{fg}$
	24	3.93 ± 0.07 ${}^{bcdef}$	5.90 ± 0.13 ${}^{efg}$	n.d.	4.02 ± 0.17 ${}^{de}$	13.15 ± 0.14 ${}^{efg}$
	30	4.10 ± 0.08 ${}^{abcdef}$	5.99 ± 0.07 ${}^{ef}$	2.52 ± 0.06 ^*e*^	4.24 ± 0.153 ${}^{cd}$	18.32 ± 1.20 ${}^{cde}$
	36	4.40 ± 0.10 ${}^{abc}$	6.41 ± 0.24 ${}^{def}$	2.78 ± 0.07 ${}^{de}$	4.64 ± 0.15 ${}^{bc}$	21.27 ± 2.16 ${}^{c}$
	42	4.54 ± 0.11 ${}^{a}$	6.69 ± 0.20 ${}^{cde}$	2.94 ± 0.03 ${}^{de}$	5.05 ± 0.26 ${}^{ab}$	24.02 ± 2.61 ${}^{bc}$
	48	4.60 ± 0.08 ${}^{a}$	6.45 ± 0.27 ${}^{def}$	2.99 ± 0.07 ${}^{de}$	5.22 ± 0.18 ${}^{a}$	29.40 ± 5.30 ${}^{ab}$
Faba bean	0	4.22 ± 0.53 ${}^{abcde}$	n.d.	n.d.	3.15 ± 0.24 ${}^{gh}$	n.d.
high-protein	6	4.15 ± 0.12 ${}^{abcdef}$	3.60 ± 0.85 ${}^{h}$	n.d.	3.86 ± 0.21 ${}^{def}$	3.38 ± 0.08 ${}^{h}$
flour	12	3.20 ± 0.43 ${}^{g}$	5.46 ± 0.60 ${}^{fg}$	n.d.	2.95 ± 0.29 ${}^{h}$	7.94 ± 0.98 ${}^{gh}$
(PRSD)	18	4.23 ± 0.13 ${}^{abcd}$	7.77 ± 0.14 ${}^{bc}$	3.95 ± 0.21 ${}^{cd}$	3.69 ± 0.08 ${}^{defg}$	14.66 ± 0.66 ${}^{def}$
	24	3.75 ± 0.31 ${}^{defg}$	7.42 ± 0.63 ${}^{bcd}$	4.58 ± 0.43 ${}^{bc}$	3.65 ± 0.25 ${}^{efg}$	17.94 ± 2.42 ${}^{cde}$
	30	4.27 ± 0.17 ${}^{abcd}$	8.96 ± 0.64 ${}^{a}$	5.26 ± 0.71 ${}^{ab}$	4.11 ± 0.08 ${}^{cde}$	20.30 ± 1.84 ${}^{cd}$
	36	3.83 ± 0.50 ${}^{cdef}$	8.36 ± 1.02 ${}^{ab}$	5.26 ± 0.99 ${}^{ab}$	3.98 ± 0.47 ${}^{de}$	21.26 ± 3.21 ${}^{c}$
	42	4.43 ± 0.43 ${}^{ab}$	9.15 ± 0.88 ${}^{a}$	5.89 ± 0.94 ${}^{a}$	4.97 ± 0.38 ${}^{ab}$	28.25 ± 3.22 ${}^{ab}$
	48	4.23 ± 0.27 ${}^{abcd}$	9.06 ± 0.45 ${}^{a}$	6.27 ± 1.03 ${}^{a}$	5.14 ± 0.48 ${}^{ab}$	33.54 ± 7.12 ${}^{a}$

Means ± standard deviation with different lower case letters in the same column are significantly different at *p* < 0.05. n.d., not detected or levels below 0.02 %DM. ${}^{S}$ Two-way ANOVA confirmed significant effect (*p* < 0.05) of substrate (DF/PR) on this variable. ${}^{T}$ Two-way ANOVA confirmed significant effect (*p* < 0.05) of fermentation time (0–6 h) on this variable. ${}^{S:T}$ Two-way ANOVA confirmed significant interactive effect (*p* < 0.05) of substrate and fermentation time on this variable.

**Table 5:** Flour properties of wheat flour (used for control) and faba bean flour mixes (used for faba bean formulations).

Variable	Wheat Flour	DF Flour Mix	DFSD Flour Mix	PR Flour Mix	PRSD Flour Mix
**Farinograph**					
Farinograph water absorption					
(FWA) ${}^{*}$ [%]	60.57 ± 0.09 ${}^{c}$	60.73 ± 0.09 ${}^{c}$	61.27 ± 0.05 ${}^{b}$	59.87 ± 0.12 ${}^{d}$	62.43 ± 0.17 ${}^{a}$
**GlutoPeak**					
Peak maximum time					
(PMT) [s]	45.7 ± 1.1 ${}^{b}$	58.5 ± 1.2 ${}^{a}$	36.7 ± 1.8 ${}^{c}$	29.7 ± 0.8 ${}^{d}$	32.0 ± 0.7 ${}^{d}$
Torque maximum					
(TM) [BU]	77.0 ± 0.0 ${}^{a}$	49.7 ± 1.1 ${}^{d}$	56.7 ± 0.4 ${}^{bc}$	54.7 ± 0.4 ${}^{c}$	58.3 ± 1.1 ${}^{b}$

* Measured water absorption corrected for water amount present in faba bean fermentates (DFSD and PRSD). Means ± standard deviation with different lower case letters in the same row are significantly different at *p* < 0.05.

**Table 6:** Bread quality characteristics of control wheat bread and faba bean bread formulations.

Variable	CWB	DFB	DFSDB	PRB	PRSDB
pH of dough	5.76 ± 0.04 ${}^{a}$	5.79 ± 0.07 ${}^{a}$	5.47 ± 0.02 ${}^{b}$	5.77 ± 0.02 ${}^{a}$	5.69 ± 0.06 ${}^{a}$
Bake loss [%]	12.0 ± 0.7 ${}^{b}$	11.4 ± 0.5 ${}^{c}$	12.8 ± 0.9 ${}^{a}$	10.6 ± 0.9 ${}^{d}$	12.2 ± 0.9 ${}^{b}$
Specific volume (SV) [ml/g]	3.09 ± 0.12 ${}^{a}$	2.42 ± 0.09 ${}^{d}$	2.96 ± 0.10 ${}^{b}$	2.16 ± 0.07 ^*e*^	2.58 ± 0.09 ${}^{c}$
Hardness day 0 [N]	6.31 ± 1.01 ^*e*^	15.65 ± 1.51 ${}^{b}$	8.02 ± 1.01 ${}^{d}$	18.50 ± 1.4 ${}^{a}$	11.44 ± 1.78 ${}^{c}$
Hardness day 2 [N]	13.04 ± 1.46 ${}^{d}$	30.41 ± 4.13 ${}^{b}$	14.79 ± 1.48 ${}^{d}$	33.51 ± 2.83 ${}^{a}$	19.17 ± 2.26 ${}^{c}$
Hardness day 5 [N]	20.65 ± 2.57 ^*e*^	39.33 ± 3.26 ${}^{b}$	22.66 ± 2.59 ${}^{d}$	44.53 ± 4.99 ${}^{a}$	28.04 ± 2.84 ${}^{c}$
Staling rate [ΔN/d]	2.31 ± 0.36 ${}^{a}$	1.53 ± 0.25 ${}^{c}$	1.86 ± 0.43 ${}^{b}$	1.42 ± 0.31 ${}^{c}$	1.49 ± 0.34 ${}^{c}$
Number of cells	1527 ± 95 ${}^{a}$	1338 ± 94 ${}^{b}$	1243 ± 80 ${}^{bc}$	1454 ± 123 ${}^{a}$	1144 ± 129 ${}^{c}$
Cell area [%]	48.4 ± 1.1 ${}^{a}$	46.9 ± 1.0 ${}^{b}$	49.6 ± 1.0 ${}^{a}$	46.3 ± 1.6 ${}^{b}$	49.4 ± 1.0 ${}^{a}$
Lightness of crumb (L ${}^{*}$crumb)	76.9 ± 2.7 ${}^{a}$	74.7 ± 2.0 ${}^{b}$	71.5 ± 1.4 ${}^{cd}$	73.2 ± 1.9 ${}^{bc}$	70.6 ± 2.3 ${}^{d}$
Lightness of crust (L ${}^{*}$crust)	77.3 ± 2.6 ${}^{a}$	70.5 ± 3.8 ${}^{b}$	64.7 ± 2.5 ${}^{cd}$	63.3 ± 5.0 ${}^{c}$	61.7 ± 2.5 ${}^{d}$

Means ± standard deviation with different lower case letters in the same row are significantly different at *p* < 0.05.

**Table 7:** Values for resistant, digestible and total starch reported in %DM and hydrolysis indices (HIs) determined by in vitro starch digestion and monitoring of released reducing sugars over a time of 240 min for CWB, DFB, DFSDB, PRB and PRSDB.

Variable	CWB	DFB	DFSDB	PRB	PRSDB
**Resistant starch** [%DM]	1.18 ± 0.02 ${}^{ab}$	1.45 ± 0.16 ${}^{a}$	1.26 ± 0.00 ${}^{a}$	0.93 ± 0.04 ${}^{b}$	0.95 ± 0.04 ${}^{b}$
**Digestible starch** [%DM]	70.54 ± 0.65 ${}^{a}$	66.75 ± 0.08 ${}^{b}$	67.06 ± 0.35 ${}^{b}$	61.96 ± 0.12 ${}^{c}$	61.96 ± 1.17 ${}^{c}$
**Total starch** [%DM]	71.72 ± 0.67 ${}^{a}$	68.20 ± 0.08 ${}^{b}$	68.32 ± 0.35 ${}^{b}$	62.89 ± 0.15 ${}^{c}$	62.91 ± 1.13 ${}^{c}$
**Hydrolysis index** [%]	100 ${}^{*}$	99.9 ± 3.8 ${}^{c}$	119.0 ± 3.7 ${}^{ab}$	133.4 ± 4.4 ${}^{a}$	114.9 ± 2.3 ${}^{b}$

* HIs of DFB, DFSDB, PRB and PRSDB calculated as areas under their sugar release curves (30 to 240 min) relative to the area under CWB’s sugar release curve (30 to 240 min). Means ± standard deviation with different lower case letters in the same row are significantly different at *p* < 0.05.

## Abbreviations

The following abbreviations are used in this manuscript.

ANC	Antinutritional compound
ANOVA	Analysis of variance
BU	Brabender units
CA	Correlation analysis
CFU	Cell forming units
CWB	Control wheat bread
DF	Faba bean dehulled flour
DFB	Wheat bread containing DF
DFSD	DF fermented with *Ln. citreum TR116* starting from inoculation (0 h)
DFSDB	Wheat bread containing DFSD
DMSO	Dimethylsulfoxide
DNS	3,5-Dinitrosalicylic acid
DY	Dough yield
EPS	Exopolysaccharides
FB	Faba bean
FQ	Fermentation quotient
FU	Farinograph units
FWA	Farinograph water absorption
GI	Glycaemic index
GL	Glycaemic load
HI	Hydrolysis index

HPI	High-protein ingredient
LAB	Lactic acid bacteria
L ${}^{*}$crumb	Lightness of crumb in the CIE L ${}^{*}$a ${}^{*}$b ${}^{*}$ colour space
L ${}^{*}$crust	Lightness of crust in the CIE L ${}^{*}$a ${}^{*}$b ${}^{*}$ colour space
MRS	deMan-Rogosa-Sharpe
PMT	Peak maximum time
PR	Air-classified faba bean high-protein flour
PRB	Wheat bread containing PR
PRSD	PR fermented with *Ln. citreum* TR116 starting from inoculation (0 h)
PRSDB	Wheat bread containing PRSD
QuEChERS	quick, easy, cheap, effective, rugged and safe
RFO	Raffinose family oligosaccharides
SEM	Scanning electron microscopy
SPE	Solid phase extraction
SV	Specific volume
TM	Torque maximum
TTA	Total titratable acids
%DM	Percentage based on dry matter
